# Cortical and thalamic connections of the human globus pallidus: Implications for disorders of consciousness

**DOI:** 10.3389/fnana.2022.960439

**Published:** 2022-08-25

**Authors:** Zhong S. Zheng, Martin M. Monti

**Affiliations:** ^1^Department of Psychology, University of California, Los Angeles, Los Angeles, CA, United States; ^2^Research Institute, Casa Colina Hospital and Centers for Healthcare, Pomona, CA, United States; ^3^Brain Injury Research Center (BIRC), Department of Neurosurgery, University of California, Los Angeles, Los Angeles, CA, United States

**Keywords:** globus pallidus, diffusion tractography, basal ganglia, disorders of consciousness, arousal regulation

## Abstract

A dominant framework for understanding loss and recovery of consciousness in the context of severe brain injury, the mesocircuit hypothesis, focuses on the role of cortico-subcortical recurrent interactions, with a strong emphasis on excitatory thalamofugal projections. According to this view, excess inhibition from the internal globus pallidus (GPi) on central thalamic nuclei is key to understanding prolonged disorders of consciousness (DOC) and their characteristic, brain-wide metabolic depression. Recent work in healthy volunteers and patients, however, suggests a previously unappreciated role for the external globus pallidus (GPe) in maintaining a state of consciousness. This view is consistent with empirical findings demonstrating the existence of “direct” (i.e., not mediated by GPi/substantia nigra pars reticulata) GPe connections with cortex and thalamus in animal models, as well as their involvement in modulating arousal and sleep, and with theoretical work underscoring the role of GABA dysfunction in prolonged DOC. Leveraging 50 healthy subjects' high angular resolution diffusion imaging (HARDI) dataset from the Human Connectome Project, which provides a more accurate representation of intravoxel water diffusion than conventional diffusion tensor imaging approaches, we ran probabilistic tractography using extensive *a priori* exclusion criteria to limit the influence of indirect connections in order to better characterize “direct” pallidal connections. We report the first *in vivo* evidence of highly probable “direct” GPe connections with prefrontal cortex (PFC) and central thalamic nuclei. Conversely, we find direct connections between the GPi and PFC to be sparse (i.e., less likely indicative of true “direct” connectivity) and restricted to the posterior border of PFC, thus reflecting an extension from the cortical motor zones (i.e., motor association areas). Consistent with GPi's preferential connections with sensorimotor cortices, the GPi appears to predominantly connect with the sensorimotor subregions of the thalamus. These findings are validated against existing animal tracer studies. These findings suggest that contemporary mechanistic models of loss and recovery of consciousness following brain injury must be updated to include the GPe and reflect the actual patterns of GPe and GPi connectivity within large-scale cortico-thalamo-cortical circuits.

## Introduction

The crucial role of cortico-subcortical interactions in the maintenance of waking consciousness has long been appreciated. While the content of consciousness, the experience itself, might crucially rely on cortical sites, the thalamus is often considered to play a key role in allowing the cortex to generate the type of neural activity that enables conscious experience (Alkire and Miller, 2005). Damage to the thalamus is believed to be among the most prevalent signatures of protracted unconsciousness in postmortem studies (Adams et al., 1999, 2000; Graham et al., 2005). Moreover, *in vivo* measures of thalamic damage and atrophy (Fernández-Espejo et al., 2011; Lutkenhoff et al., 2015), as well as its deafferentation from the cortex (Lant et al., 2016; Zheng et al., 2017), have been shown to be proportional to the depth of the impairment in patients with chronic disorders of consciousness (DOC). Conversely, thalamic stimulation has been shown to produce awakening from anesthesia in animal models (Alkire et al., 2007, 2009; Yoo et al., 2011; Redinbaugh et al., 2020; Bastos et al., 2021) and clinically observable improvements in patients with DOC (Schiff et al., 2007; Cain et al., 2021).

In line with this work, a mesocircuit mechanism has been proposed to explain the loss and recovery of consciousness following severe brain injury (Schiff, 2010). Under this view, DOCs emerge as a circuit-level dysfunction characterized by a global decrease in synaptic background activity. Two mechanisms are posited to contribute to this dysfunction. First, a unique vulnerability of central thalamic nuclei (i.e., intralaminar complex and adjacent paralaminar portion of association nuclei—mediodorsal, ventral anterior, ventral lateral, and pulvinar), due to their widespread point-to-point connections with the cortex (Scannell et al., 1999), makes them particularly susceptible to deafferentation following brain injury (Maxwell et al., 2006; Schiff, 2008, 2016), thus leading to a decrease in thalamopetal and striatopetal excitatory outputs. Second, compounding this effect, reduced cortico-striatal and thalamo-striatal excitatory inputs decrease the activity of the striatal medium spiny neurons (Grillner et al., 2005), thereby releasing the GPi from their GABAergic input and allowing it to actively inhibit thalamic neurons, further depressing its glutamatergic output (cf., Schiff, 2010, 2016, for further details).

A growing body of evidence suggests that the external globus pallidus (GPe) might also be an important node in the maintenance of electrocortical and behavioral arousal. In the rodent model (Qiu et al., 2010, 2016a,b; Vetrivelan et al., 2010; Yuan et al., 2017), optogenetic and deep brain stimulation (DBS) of the GPe has been shown to increase sleep and EEG delta power (Qiu et al., 2016a,b), while cell-body-specific lesion of GPe neurons increased diurnal wake and decreased diurnal non-REM sleep (Qiu et al., 2010). Moreover, the sleep-promoting adenosine A_2A_ receptors found densely in the striatum have been shown to innervate parvalbumin (PV) neurons of the rostral GPe (Yuan et al., 2017). In contrast to the findings in the GPe, lesions of the GPi do not appear to alter sleep–wake activity (Qiu et al., 2010). Mirroring these data, GPe dysfunction is also believed to be an important component of the sleep symptomatology in Parkinson's disease (Albers et al., 2017). Indeed, pharmacological restoration of GPe function (Trenkwalder, 1998) and electrical stimulation of the GPe (Castillo et al., 2020) have been shown to relieve sleep symptoms in this patient cohort.

Intriguingly, recent work has also shown an association between the degree of atrophy in the globus pallidus, and its lateral aspect in particular, and neurobehavioral (Lutkenhoff et al., 2015) and neurophysiological (Lutkenhoff et al., 2020b) biomarkers of impairment in DOC, in line with the results reported in the rodent model (Qiu et al., 2010). Furthermore, GPe atrophy in a cohort of DOC patients was also found to be the only region correlated (inversely) with a well-validated quantitative measure associated with a state of conscious awareness (i.e., the perturbational complexity index) (Lutkenhoff et al., 2020a).

As the second largest component of the basal ganglia (BG), just after the striatum, GPe not only contains extensive connections with BG structures but also direct connections with the frontal cortex (Chen et al., 2015; Saunders et al., 2015) and thalamus (Hazrati and Parent, 1991; Chattopadhyaya and Pal, 2004; Mastro et al., 2014). As reviewed above, direct GABAergic output from GPe to the frontal cortex (Vetrivelan et al., 2010; Chen et al., 2015) may be an important pathway for sleep–wake regulation (Qiu et al., 2010; Yuan et al., 2017). Consistent with this line of evidence, a recent study of effective connectivity in healthy volunteers undergoing different levels of anesthesia (i.e., wake, sedation, loss of consciousness) shows that the addition of a “direct” pallido-frontal link to the traditional mesocircuit model better explains the data, with this specific link fading with loss of consciousness and returning with its recovery (Crone et al., 2017).

Thus far, “direct” GPe connections with structures outside of the basal ganglia have been verified in animals, but are not well-characterized in humans, likely due to the obsolete nature of postmortem methods. Diffusion-based tractography, while suffering from several limitations (e.g., inability to differentiate between afferent and efferent connections, difficulty in teasing apart direct and indirect connections, susceptibility to false-positive and false-negative connections, etc.), remains the only non-invasive technique for delineating anatomical connectivity in the human brain *in vivo*. While previous studies (Milardi et al., 2015; Grewal et al., 2018) have attempted to characterize the connections between the pallidum and cortex using diffusion-based tractography to suggest a likely existence of a “direct” corticopallidal pathway, they did not account for the influence of indirect connections, which can increase the risk of false-positive direct connections.

In this study, we aim to (i) provide tractographic evidence of the probable existence of “direct” GPe connections with cortex and thalamus in humans using extensive *a priori* exclusion criteria to minimize the influence of indirect connections and (ii) contrast the patterns of cortical and thalamic connectivity of the GPe with that of the GPi. To achieve these objectives, we employ probabilistic tractography using high angular resolution diffusion imaging (HARDI) data from the Human Connectome Project (HCP) (Van Essen et al., 2012), which offer, as compared to conventional diffusion tensor imaging (DTI) approaches, the greater advantage of resolving intravoxel fiber heterogeneity, therefore providing higher spatial and angular resolution (Tuch et al., 2002) and thus improving the accuracy of tractography (Berman et al., 2013; Thomas et al., 2014). Furthermore, the implementation of probabilistic tractography, as opposed to deterministic tractography, enables increased sensitivity in detecting additional fiber tracts that may not be part of major white matter pathways, thus allowing for more exploratory fiber tracking, especially in delineating novel pathways. Incorporating *a priori* exclusion criteria helps to limit indirect connections, so that our results can more accurately reflect “direct” connections only. Given the individual variability in seed and target sizes, normalizing and rescaling the results would permit for more faithful quantitative comparison across subjects.

For terminology clarification, since diffusion tractography cannot distinguish afferent and efferent connections, the terms we use to describe connectivity between two regions (e.g., pallidocortical, pallidothalamic) do not indicate directionality; the former (“pallido”) merely reflects the seed region and the latter (“cortical” or “thalamic”) the target region.

## Methods

### Data

We analyzed HARDI data provided by the HCP (Q3 Release), WU-Minn Consortium (http://www.humanconnectome.org) (Van Essen et al., 2012). Fifty healthy subjects (26 females; age 22–35) were included in the analysis. Imaging data were acquired on a modified 3T Siemens Skyra scanner. T1-weighted structural (TR = 2,400 ms; TE = 2.14 ms; flip angle = 8°; FOV = 224 × 224 mm; voxel size = 0.7 mm isotropic) and diffusion-weighted HARDI (TR = 5,520 ms; TE = 89.5 ms; flip angle = 78°; FOV = 210 × 180 mm; voxel size = 1.25 mm isotropic; 3 shells of b = 1,000, 2,000, and 3,000 s/mm^2^ with 90 diffusion directions per shell) data were used. Basic diffusion preprocessing steps have been applied which included B0 image intensity normalization, EPI distortion correction, eddy current correction, motion correction, gradient non-linearity correction, and registration of diffusion data with T1-weighted structural data (Glasser et al., 2013). Preprocessing was accomplished using FSL tools (http://fsl.fmrib.ox.ac.uk).

### Regions of interest (ROIs)

**Figure 1:**
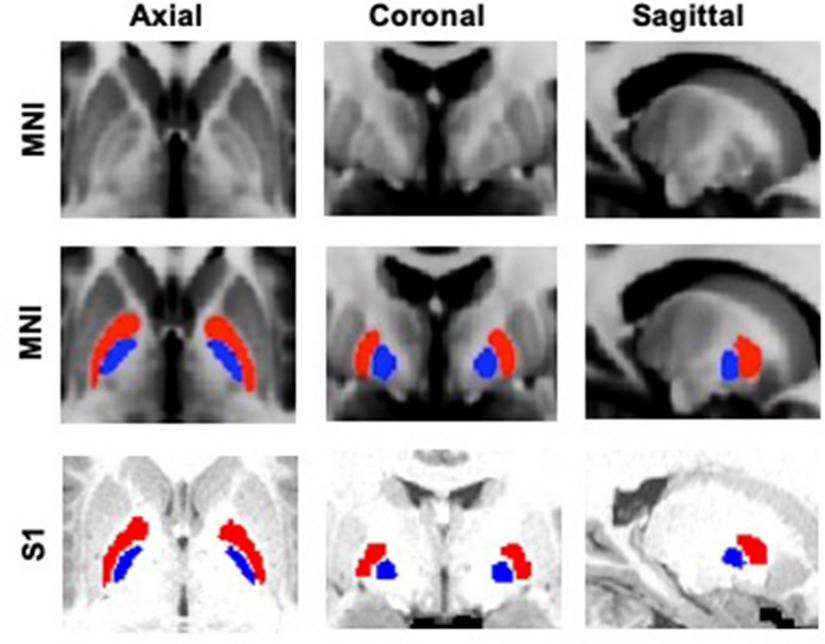
GPe (red) and GPi (blue) ROIs overlaid on standard MNI (Montreal Neurological Institute) 1 mm template and a sample subject's (S1) T1 image.

For cortical and thalamic ROIs, segmented FreeSurfer labels supplied by the HCP were used (http://surfer.nmr.mgh.harvard.edu). To create the five distinct cortical zones [prefrontal (PFC), sensorimotor (SMC), posterior parietal (PPC), temporal (TEM), and occipital (OCC)], selected Desikan–Killiany atlas labels were combined together (Supplementary Table 1, Supplementary Figure 1) (Desikan et al., 2006). Moreover, brainstem (BrS) and cerebellum (CB) masks were also obtained from FreeSurfer. Basal ganglia ROIs, including GPe, GPi, striatum (STR), substantia nigra (SN), and subthalamic nucleus (STN), were extracted from the Keuken and Forstmann's 7T atlas (Keuken and Forstmann, 2015), which has been used as part of established toolboxes to facilitate DBS electrode reconstructions (lead-dbs.org). A threshold of 50% was applied to all the BG masks, except for STN, which was thresholded at 25% due to its small size. The BG masks were first resized to Montreal Neurological Institute (MNI) 1 mm resolution and then transformed into each subject's native T1 space using FLIRT (Jenkinson et al., 2002). All masks in T1 space were subsequently aligned, with affine registration, to each individual's native diffusion space. To refine the GPe and GPi masks, we used the segmented whole pallidum mask from FreeSurfer to constrain the boundaries. GPe and GPi ROIs are shown in Figure 1.

### Probabilistic tractography

**Figure 2:**
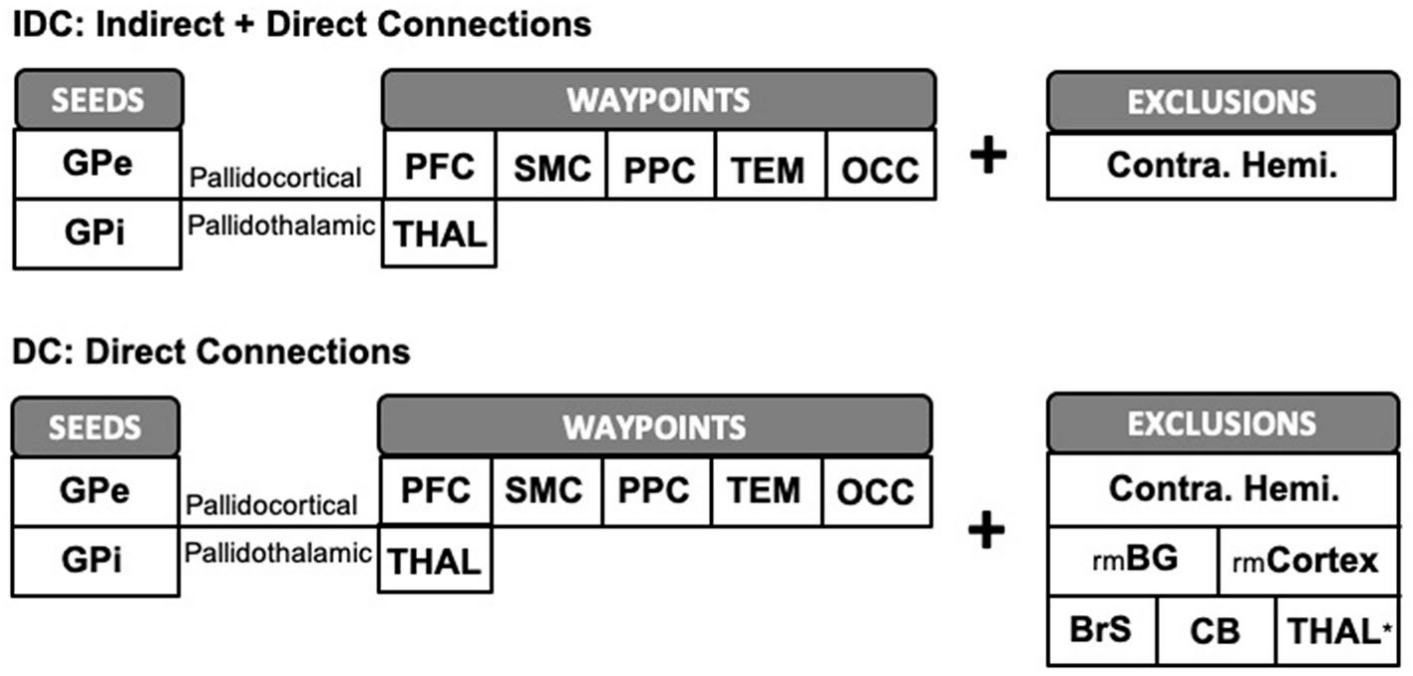
Schematic representation of pallidocortical and pallidothalamic tractography workflow. We ran probabilistic tractography twice, both times limiting the tractography ipsilaterally by supplying an exclusion mask of the contralateral hemisphere. The first procedure, IDC, was carried out with GPe/GPi each serving as a seed ROI with cortical or thalamic ROIs as waypoints, without being restricted by additional exclusion criteria. To limit the influence of indirect connections, we repeated tractography with additional exclusion criteria to better reflect direct pallidal connections (DCs). GPe, external globus pallidus; GPi, internal globus pallidus; PFC, prefrontal; SMC, sensorimotor; PPC, posterior parietal; TEM, temporal; OCC, occipital; THAL, thalamus; rmBG, remaining basal ganglia regions; rmCortex, remaining cortical regions; BrS, brainstem; CB, cerebellum; Contra. Hemi., contralateral hemisphere. *N/A for pallidothalamic tractography.

Imaging analyses were accomplished using FSL tools. First, the probability distribution function of fiber orientations at each voxel was estimated with the following parameters: 3 fibers per voxel, 1,000 burn-ins, deconvolution model using zeppelins, and gradient non-linearities considered. Next, probabilistic tractography was launched with 5,000 samples drawn per voxel from each seed mask. For pallidocortical and pallidothalamic connectivity analyses, GPe and GPi each served as a seed mask with each of the five cortical targets and thalamus (THAL) as a waypoint mask, per hemisphere (Figure 2). A contralateral hemispheric mask was included as an exclusion criterion to limit the connections ipsilaterally. This procedure generated ipsilateral pallidocortical and pallidothalamic connections, though indirect connections arising from other nearby structures were also considered. For ease of referencing, we will refer to the results of this step as “indirect and direct” connections (IDCs). To limit the influence of indirect connections, we repeated probabilistic tractography with additional exclusion criteria based on *a priori* knowledge of basal ganglia connections with cortico-subcortical circuits, whereby streamlines entering any of the following masks were discarded: the remaining BG (STR, SN, STN, and GPe or GPi) and cortical ROIs, thalamus, brainstem, cerebellum, in addition to the contralateral hemisphere. The results from this analysis will be referred to as “direct connections” (DCs) only. As a final analysis to more precisely visualize the different subregions within thalamus that GPe and GPi may be directly connected with, the thalamus also served as a seed with GPe and GPi as targets, following similar exclusion criteria. Next, the number of streamlines (i.e., connectivity strengths) that successfully reached the cortical and thalamic targets from the pallidal seeds was computed after applying a threshold of 50 (1% of 5,000 samples per voxel) to remove spurious connections for both IDC and DC. The results were then normalized and rescaled to account for individual differences in seed and target sizes (Eickhoff et al., 2010). Individual streamline counts were divided by the total number of samples sent (5,000 × seed mask size) and then rescaled by multiplying by the average of all total number of samples sent across all seeds and targets. This step accounted for differences in seed sizes. Next, the resulting values were then divided by the size of the targets and then again multiplied by the average size of all targets. This accounted for the variability in target sizes. For display of the tractograms, a threshold of 0.01% of the total number of streamlines sent was applied with an additional threshold of least 10% of subjects (*n* = 5) sharing the tracks. To identify the thalamic subregion containing the highest GPe or GPi connectivity, we used fslstats function on the normalized average group connectivity maps to find the coordinates of the maximum voxel. The Morel thalamic atlas (Niemann et al., 2000) was used as an anatomical reference to determine the subregional location of pallidal connectivity within thalamus.

### Statistical analysis

Repeated measures ANOVAs of normalized and rescaled pallidal connectivity values were carried out for IDC and DC with seed (GPe, GPi), target (PFC, SMC, PPC, TEM, OCC, THAL), and hemisphere (left, right) as within-group factors. Upon finding significance, pairwise *t*-tests comparing GPe and GPi connections were performed along with multiple comparisons correction using the Benjamini and Hochberg (1995) method with false discovery rate set at 0.05. The new significance cutoff value was thus established at *p* < 0.02. Moreover, the percent change of total streamline counts from IDC to DC was calculated.

## Results

### Assessing pallidocortical and pallidothalamic connectivity strengths

**Figure 3:**
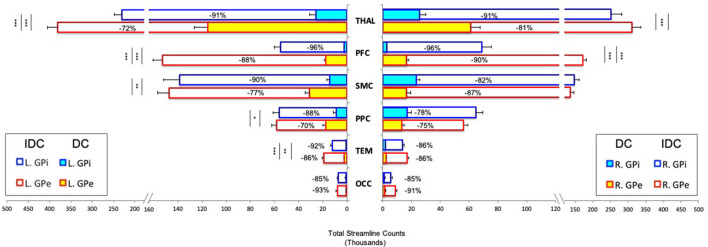
Pallidocortical and pallidothalamic connectivity strengths as assessed by the group averages of total streamline counts between each seed (GPe/GPi) and target ROI (THAL/PFC/SMC/PPC/TEM/OCC) are plotted. Direct connections (DCs) and indirect and direct connections (IDCs) are overlapped to reflect a percent decrease from IDC to DC. GPe and GPi connections are statistically compared, with significance denoted using inner asterisks to correspond to DC and outer asterisks to IDC. Error bars reflect S.E.M. *p < 0.05, **p < 0.01, ***p < 0.001. GPe, external globus pallidus; GPi, internal globus pallidus; THAL, thalamus; PFC, prefrontal; SMC, sensorimotor; PPC, posterior parietal; TEM, temporal; OCC, occipital; L, left; R, right.

The total number of streamlines (normalized and rescaled) that successfully reached the cortical and thalamic targets from the pallidal seeds for both IDC and DC is plotted in Figure 3. Statistical results are reported below:

IDC: Significant main effects of seed (*F* = 109.063, *p* < 0.0001) and target (*F* = 153.47, *p* < 0.0001) and significant interactions of hemisphere × seed × target (*F* = 7.7, *p* < 0.01), hemisphere × seed (*F* = 11.2, *p* < 0.01), and seed × target (*F* = 46.9, *p* < 0.0001) were observed. Comparing GPe and GPi connectivity, *post-hoc t*-tests uncovered significantly higher connections for bilateral GPe-PFC, left GPe-THAL, and GPe-TEM than GPi with these targets.

DC: Significant main effects of seed (*F* = 59.6, *p* < 0.0001), target (*F* = 106.5, *p* < 0.0001), and hemisphere (*F* = 15, *p* < 0.001) along with significant interactions of hemisphere × seed × target (*F* = 15.8, *p* < 0.0001), hemisphere × seed (*F* = 34.5, *p* < 0.0001), hemisphere × target (*F* = 18.9, *p* < 0.0001), and seed × target (*F* = 51.7, *p* < 0.0001) were detected. *Post-hoc* comparisons revealed significantly greater connections for bilateral GPe-PFC and GPe-THAL, left GPe-SMC, GPe-PPC, and GPe-TEM than GPi with these targets.

Overall, while the general pattern of DC remained roughly the same as IDC after introducing comprehensive exclusion criteria to minimize the influence of indirect connections, the direct connectivity values reduced drastically, with up to 96% decrease from IDC to DC, with GPi appearing to suffer more than GPe for most of the connections (Figure 3). We evaluated relative residual direct connectivity strength by separating the total streamline counts (tSC) into three categories: high (tSC > 10,000), medium (5,000–10,000 tSC), and low (tSC <5,000) probability of connection. THAL, SMC, and PPC exhibited a medium to high probability of direct connections with GPe and GPi, but only GPe, not GPi, demonstrated a high likelihood of direct connection with PFC. A low probability of direct GPe/GPi connectivity with TEM and OCC was found.

### Topographical organization of GPe and GPi connections

**Figure 4:**
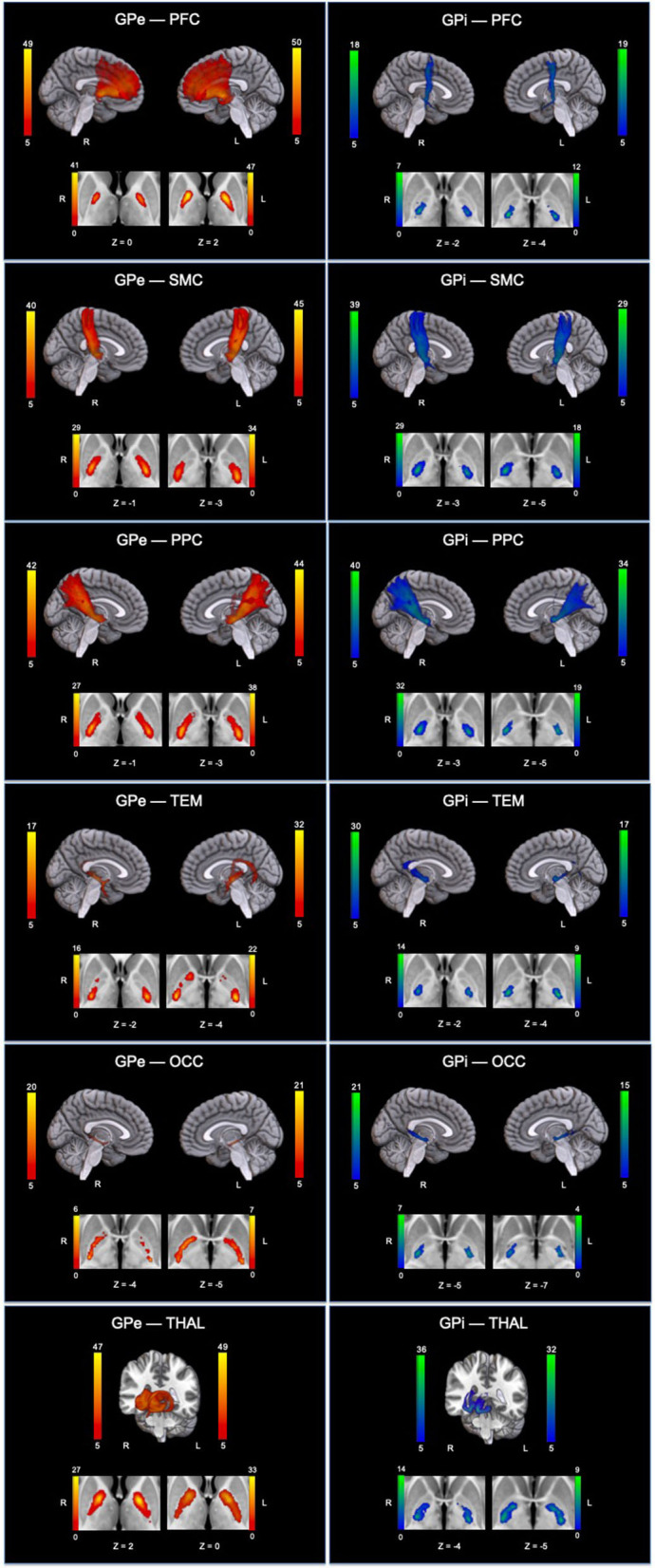
Topographical patterns of direct pallidal connectivity are depicted. Left column displays GPe connections (red-yellow), and right column displays GPi connections (blue-green). For each panel, the top section shows reconstructed pallidal tracks, and cropped axial images below depict preferred subregional connectivity originating from GPe or GPi. Color bars reflect the number of subjects overlapping (max = 50 subjects). GPe, external globus pallidus; GPi, internal globus pallidus; THAL, thalamus; PFC, prefrontal; SMC, sensorimotor; PPC, posterior parietal; TEM, temporal; OCC, occipital; L, left; R, right.

**Figure 5:**
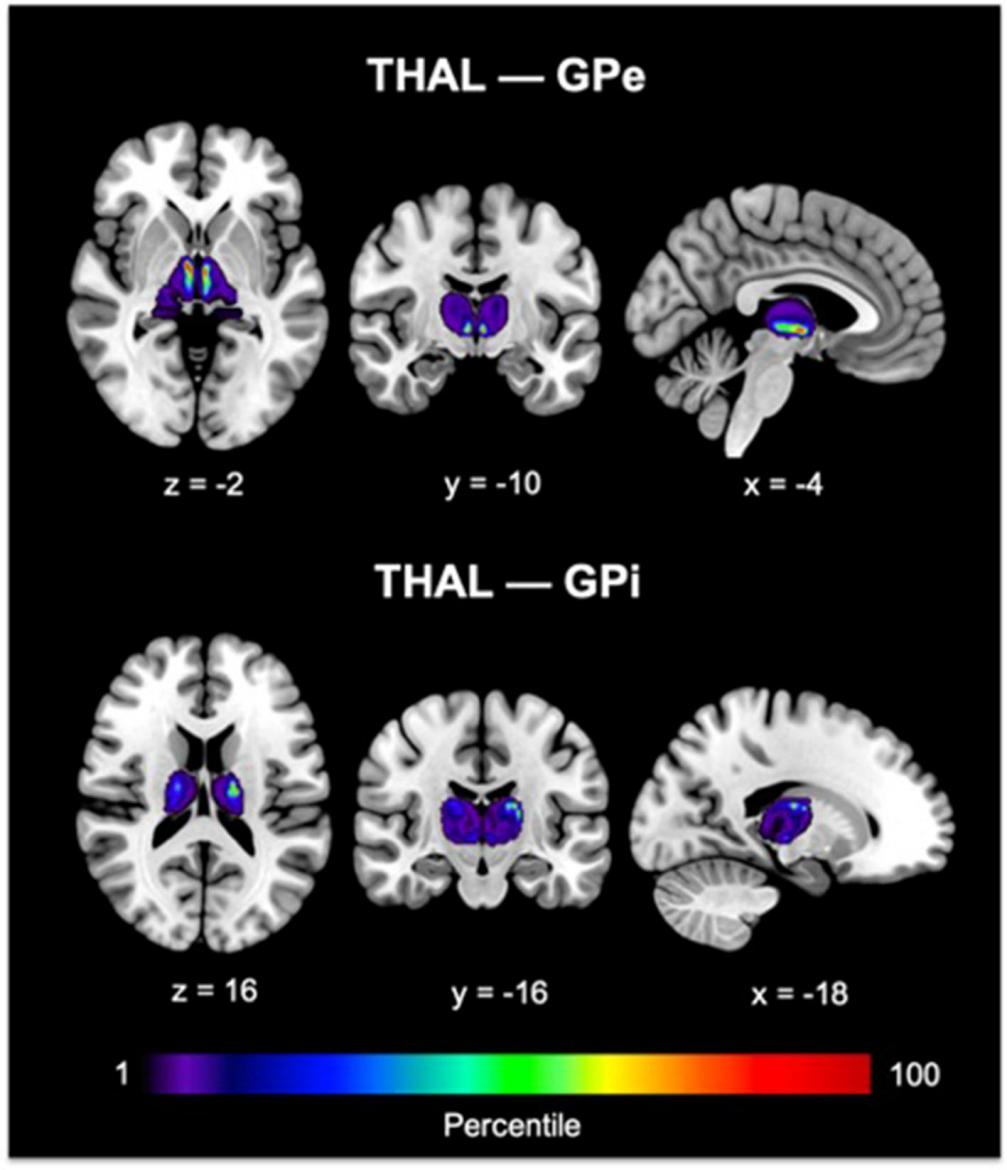
GPe and GPi connectivity within thalamus are shown. Color bars reflect the percentile of connections. For THAL—GPe connections, peak voxel was found in the central medial intralaminar nucleus. For THAL—GPi connections, peak voxel was identified in the ventral lateral thalamus. The Morel thalamic atlas was used as an anatomical guide. GPe, external globus pallidus; GPi, internal globus pallidus; THAL, thalamus.

Since our primary interest lies in direct connections, the descriptions hereafter will pertain only to DC. Group pallidal connections with the different cortical and thalamic targets and corresponding pallidal seed voxels with robust target connectivity are displayed in Figure 4. Similar topographical connectivity patterns were identified for GPe and GPi, except for PFC and THAL connections. While GPe-PFC connections covered the entire prefrontal target and originated from pallidal voxels concentrated mostly in the anterior subregion of GPe, GPi-PFC connections mainly encompassed the posterior border of PFC (presumably, association motor cortices) and localized within the posterior subregion of GPi. Similar subregions of the GPe (anterior and a small posterior cluster) and GPi (posterior) corresponded to strong connections with THAL. Furthermore, SMC, PPC, TEM, and OCC connections resided primarily in posterior portions of GPe and GPi. Given the difficulty of dissociating preferential targeting in the thalamus from individual pallidothalamic tracks, connectivity-based segmentation of THAL with respect to GPe and GPi revealed distinct patterns of disparate pallidal organization within thalamus (Figure 5). Namely, GPe connections occupied more medial aspects of thalamus, predominantly including putative midline, mediodorsal (MD), intralaminar (IL), and ventral anterior (VA) nuclei, with the highest concentration in the central medial intralaminar nucleus (CeM); GPi connections, on the other hand, were found in more lateral and posterior portions of thalamus, reflecting primarily ventral lateral (VL), ventral posterior lateral (VPL), ventral posterior medial (VPM), lateral posterior (LP), pulvinar (PUL), as well as some IL nuclei, with peak connections in VL motor thalamus. While both pallidal connections within THAL covered intralaminar nuclei, comparatively, GPe was more connected with central lateral (CL), central medial (CeM), and parafascicular (Pf) nuclei, and GPi with centromedian (CM).

## Discussion

We used probabilistic tractography with comprehensive *a priori* exclusion criteria to characterize the pattern of direct pallidal connectivity with cortex and thalamus, separating its internal and external compartments. Overall, we report two main findings. First, in line with animal work (Mastro et al., 2014; Chen et al., 2015; Saunders et al., 2015), we report the likely *in vivo* existence, in the human brain, of direct connections between the GPe and both cortex and thalamus—that is, connections not mediated by the GPi/SNr nodes. Second, we show that the two compartments of the GP map onto different patterns of cortical and thalamic connectivity. A strong, widespread coverage of GPe tracks into the PFC, as compared to the weak GPi tracks, which were restricted to the posterior border of PFC (presumably, a partial association motor area). These pallido-PFC tracks also originated from different subregions of the pallidum, with anterior GPe corresponding to GPe-PFC connections and posterior GPi representing GPi-PFC connections. Consistent with our findings, the anterior aspects of the GPe have been reported to connect with prefrontal cortex (François et al., 2004; Grabli et al., 2004), whereas the posterior portion of GPi has been well-established as a sensorimotor region (Visser-Vandewalle et al., 2009). While the strong, direct GPe-PFC connections would still survive in our analysis with a more stringent thresholding, the weak, direct GPi-PFC connectivity would most likely fail. This is consistent with direct GPe-PFC connections being validated against animal tracer studies, making them indeed likely to exist, and direct GPi-PFC connections being—if at all present—more limited and restricted to the motor regions of the frontal cortex. Indeed, GPi has been speculated to project directly to the supplementary motor area (SMA) and pre-SMA from a primate tracing study (Akkal et al., 2007; Milardi et al., 2015).

The uneven pattern of cortical connectivity is also consistent with the pallidal connectivity-based segmentation of thalamus. Indeed, the GPe displayed a greater preference for medial thalamus, including the main prefrontal projecting thalamic nucleus—MD (Klein et al., 2010) and also a part of the central thalamus and the central medial nucleus, a component of the anterior intralaminar complex. These are the very regions consistently implicated in *postmortem* (Adams et al., 1999, 2000; Graham et al., 2005) and *in vivo* (Fernández-Espejo et al., 2011; Lutkenhoff et al., 2015, 2020c). Receiving inputs from the brainstem and basal forebrain, the anterior intralaminar and mediodorsal thalamus have been postulated to play a key role in arousal regulation and possibly extending to awareness (Schiff, 2008). On the other hand, GPi was most connected with ventral lateral thalamic nucleus, implying a more motor-related function, consistent with the classic role of GPi.

Finally, we also found additional probable direct connections for both pallidal structures with SMC and PPC, although there currently lacks concrete evidence from animal or postmortem studies to fully validate these findings. These pallidocortical connections originated from the posterior portions of the pallidum, which is consistent with DTI findings in humans from Draganski et al. (2008), even though the authors did not use exclusion criteria to limit indirect connections. However, while quantitatively different, direct connections (DCs) vs. indirect and direct connections (IDCs) may remain qualitatively similar, sharing the same topographical patterns of connectivity.

Overall, these findings suggest a duality between a more frontal-focused GPe circuit, linking GPe to the PFC both directly and through the central nuclei of thalamus, and a more sensory/motor-focused GPi circuit.

Given the structural nature of our methodology, the functional role of direct GPe-PFC and GPe-thalamic pathways, in humans and DOC patients in particular, has to remain speculative. Nonetheless, the reviewed findings across animal models (Qiu et al., 2010, 2016a,b; Yuan et al., 2017), healthy volunteers (Crone et al., 2017), and Parkinson's disease (Trenkwalder, 1998; Albers et al., 2017; Castillo et al., 2020) and DOC patients (Lutkenhoff et al., 2015, 2020a,b) suggest an involvement in the regulation of electrocortical and behavioral arousal.

So, what do the present findings mean with respect to theories of disorders of consciousness? As explained above, the dominant view proposes an important role of the basal ganglia in DOC, but focuses on the excessive tonic inhibition of central thalamic nuclei due to GPi disinhibition (Schiff, 2010), and its effect on the modulation of thalamo-prefrontal mechanisms necessary for sustaining organized behavior during wakefulness (Schiff, 2008). This model, however, is an idealization that preceded much of the recent characterization of the rich neuroanatomy of the GP. In this respect, our findings support the notion that the GPe, given its connectivity, might be more implicated in modulating central thalamic nuclei, as compared to the GPi. Furthermore, in as much as frontal cortices play a key role in forebrain arousal regulation (Schiff, 2008), the present data suggest that the model might be incomplete without the inclusion of direct connections linking the external segment of the GP and prefrontal cortex. Under this extended mesocircuit model, deficits in behavioral arousal (Lutkenhoff et al., 2015) and slowing of EEG rhythms (Lutkenhoff et al., 2020b) would map onto the GPe-cortical axis, in parallel to the results in animal models (Qiu et al., 2010). Similarly, this extended model would allow interpreting the finding of direct pallido-frontal effective connectivity being modulated by loss and recovery of consciousness (Crone et al., 2017) in healthy volunteers. The extended model would also recast the interpretation of how the administration of zolpidem, a selective GABA-ω1 (a subunit of GABA-A receptor complex) agonist commonly used to treat insomnia, leads to paradoxical awakening in DOC patients (Sutton and Clauss, 2017). This effect has been proposed to be mediated by inhibition of GPi neurons, *via* action on GABA-A receptors, and thus the release of central thalamic neuron from excess inhibition (Schiff, 2010). However, considering that GPe also houses an abundance of GABA-A receptors (Xue et al., 2010) and has direct connections with frontal-projecting thalamic nuclei (i.e., central thalamus), zolpidem's action might be partially mediated by the external pallidal compartment. This interpretation could also explain the observed restoration of prefrontal activity in DOC patients responding to this intervention (Clauss et al., 2000; Brefel-Courbon et al., 2007; Williams et al., 2013; Chatelle et al., 2014). We observe that incorporating the connectivity of the GPe with both GABAergic cortical interneurons and layer V pyramidal cells (Vetrivelan et al., 2010) in the mesocircuit might allow subsuming within the model views emphasizing the brain-wide consequences of GABA dysfunction in DOC (Pistoia et al., 2014) beyond inhibition of thalamic output. Finally, the direct projection from GPe to the frontal cortex has been demonstrated, in animal models, to contain mostly GABAergic neurons, yet a subset of them may also be cholinergic (Saunders et al., 2015). GABAergic-cholinergic projection neurons are found in and around the GPe (some arising from the neighboring nucleus basalis) (Henderson, 1997; Tkatch et al., 1998; Saunders et al., 2015). Given the proximity between GPe and nucleus basalis of the basal forebrain, a major cholinergic output that also co-releases GABA (Tkatch et al., 1998), it is possible that GPe and nucleus basalis work together to influence frontal activity and arousal. Likewise, Chen et al., also identified a direct GABAergic pallidocortical output in rodents; however, at the rostral level of GPe, which is the putative subregion projecting to the frontal regions and contains sleep-regulating neurons (Yuan et al., 2017), cholinergic neurons were sparse and GABAergic neurons predominated (Chen et al., 2015). The type and quantity of neurotransmitter expressed are likely related to the specific subregion investigated.

Beyond these observations, it is intriguing to speculate about the new understanding that an extended mesocircuit model might provide. Inclusion of the GPe and a finer parcellation of functional circuits across the two segments of the pallidum, for example, could help explain cases of dissociation between a patient's level of consciousness and their behavioral responsiveness (Owen et al., 2006; Monti et al., 2010; Edlow et al., 2017; Claassen et al., 2019). Under this extended model, the GPi-thalamic axis, along with its motor cortical targets, would be mainly involved in a patient's ability to produce voluntary behavior (e.g., Fernández-Espejo et al., 2015), whereas the GPe connectivity to thalamus and frontal cortex might be more important for maintaining the appropriate balance between excitation and inhibition that is required for the emergence of complex pattern of casual interactions among cortical neurons (Rosanova et al., 2018; Lutkenhoff et al., 2020a).

Lastly, interpretation of our findings should be mindful of the fact that using diffusion imaging to infer the existence of connections between regions presents many challenges, especially for determining “direct” connections. Currently, one of the only ways of approaching the delineation of “direct” connections relies on strategically implementing exclusion criteria based on some *a priori* knowledge, as performed here. However, this procedure presents an important tradeoff. On the one hand, not adding enough exclusions may still be subjected to the influence of indirect connections, but on the other hand, including too many exclusions may be overly stringent and lead to false negatives. Because our primary goal was to investigate the existence of “direct” connections, we opted for extensive exclusion criteria, albeit overly stringent, in hopes of removing as much of the indirect connections as possible. Though there could still exist some indirect connections in our “direct” connectivity results, the majority of which are likely addressed. More generally, the inability to differentiate afferent and efferent connections remains a major limitation of diffusion tractography, although animal tracer studies suggest bidirectional connections between GPe and PFC (Chen et al., 2015; Saunders et al., 2015; Karube et al., 2019; Abecassis et al., 2020) and between GPe and thalamus (Hazrati and Parent, 1991; Kita and Kitai, 1994; Mastro et al., 2014).

## Conclusions

We demonstrated with probabilistic tractography using HARDI data provided by the HCP that direct GPe connections with prefrontal cortex and thalamus are highly likely to exist in the human brain, consistent with animal tracer studies. These direct GPe connections also exhibited differing patterns of frontal and thalamic connectivity when compared against GPi. Favoring connections with distributed prefrontal cortex and medial thalamus, GPe is situated in a position to influence key aspects of consciousness such as the regulation of electrocortical and behavioral arousal. Conversely, GPi, preferring connections with more motor-related regions, might be more important for aspects of motor control. These findings suggest the need for integrating the mesocircuit hypothesis (Schiff, 2010, 2016) with the incorporation of the GPe, and its cortical and thalamic connections, to shed light on how the balance of dysfunction in excitatory and inhibitory mechanisms underlie disorders of consciousness.

## Data availability statement

The original contributions presented in the study are included in the article/Supplementary materials, further inquiries can be directed to the corresponding author/s.

## Ethics statement

Ethical review and approval was not required for the study on human participants in accordance with the local legislation and institutional requirements. Written informed consent for participation was not required for this study in accordance with the national legislation and the institutional requirements.

## Author contributions

ZZ: conceptualization, methodology, software, formal analysis, writing—original draft, writing—review and editing, and visualization. MM: conceptualization validation, writing—review and editing, supervision, and funding acquisition. All authors contributed to the article and approved the submitted version.

## Funding

This work was funded in part by the Tiny Blue Dot Foundation. Data were provided by the Human Connectome Project, WU-Minn Consortium (Principal Investigators: David Van Essen and Kamil Ugurbil; 1U54MH091657), funded by the 16 NIH institutes and centers that support the NIH Blueprint for Neuroscience Research and by the McDonnell Center for Systems Neuroscience at Washington University.

## Conflict of interest

The authors declare that the research was conducted in the absence of any commercial or financial relationships that could be construed as a potential conflict of interest.

## Publisher's note

All claims expressed in this article are solely those of the authors and do not necessarily represent those of their affiliated organizations, or those of the publisher, the editors and the reviewers. Any product that may be evaluated in this article, or claim that may be made by its manufacturer, is not guaranteed or endorsed by the publisher.
