# Supplementary figures and images for: Cortical and thalamic connections of the human globus pallidus: Implications for disorders of consciousness
Source: Front Neuroanat. 2022 Aug 25;16:960439. doi: 10.3389/fnana.2022.960439 (PMC9453545; doi:10.3389/fnana.2022.960439)

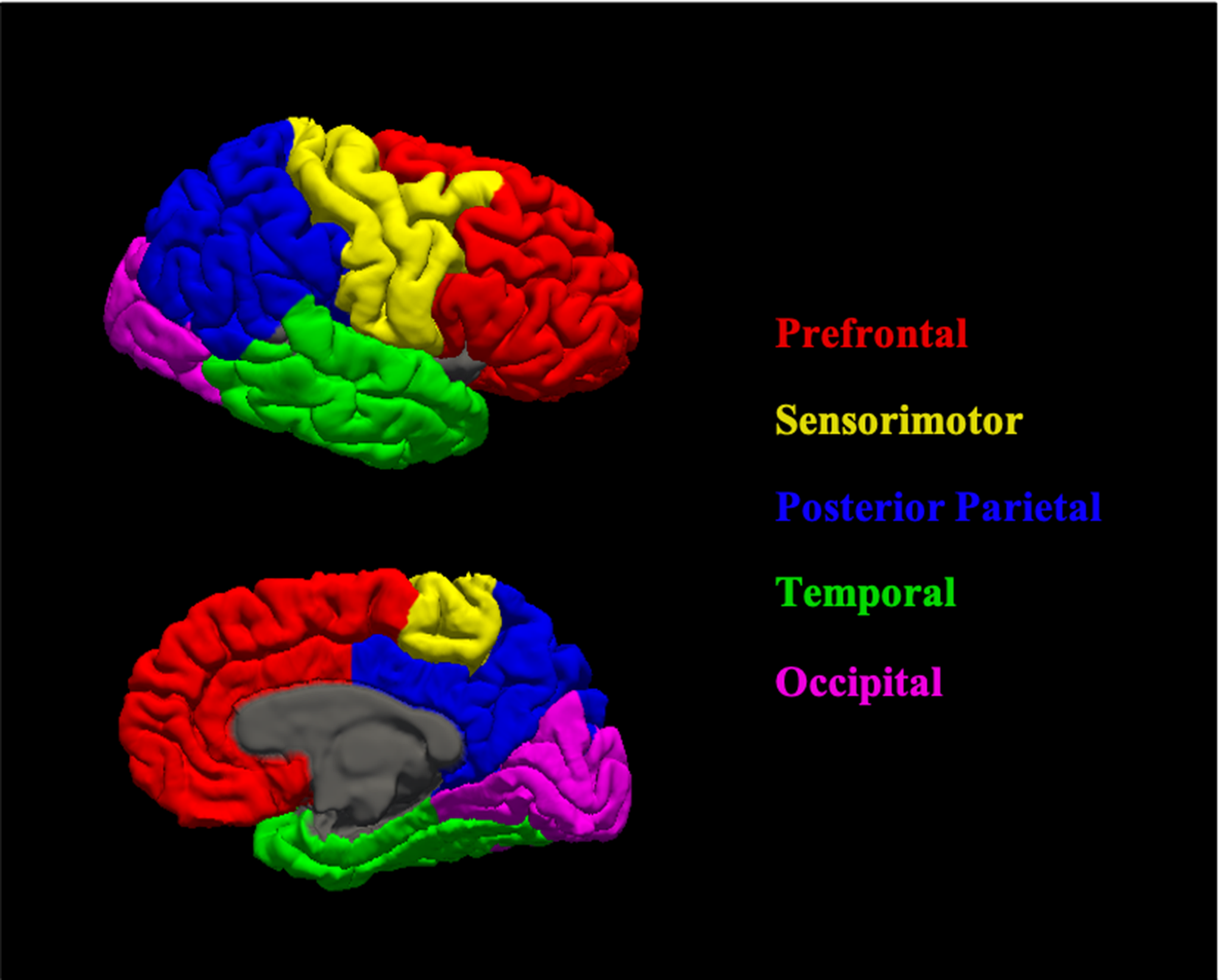

Supplement: Supplementary file 2 [file Image_1.png]
